# Concurrence of Calcium Pyrophosphate Deposition Disease in Patients Undergoing Surgery for Degenerative Spine Conditions: A Case Series and Systematic Review

**DOI:** 10.7759/cureus.105769

**Published:** 2026-03-24

**Authors:** Oluwaseyi Adebola, Masna B Inam, George Johnson, Piyali Pal, Suresh Chandrasekaran, Maggie Lee, Nisaharan Srikandarajah

**Affiliations:** 1 Neurosurgery, The Walton Centre NHS Foundation Trust, Liverpool, GBR; 2 Neuropathology, The Walton Centre NHS Foundation Trust, Liverpool, GBR

**Keywords:** calcium pyrophosphate deposition disease, spinal cppd, spinal pseudogout, spine pseudogout, spine surgery, systematic literature review

## Abstract

Background

Calcium pyrophosphate deposition disease (CPPD), also known as pseudogout, is a crystal-induced inflammatory arthropathy that commonly affects elderly individuals. Spinal involvement is considered rare or perhaps underdiagnosed. The objective of this study was to report our institutional series of patients who underwent spinal surgery for degenerative disease and whose intraoperative samples confirmed the presence of CPPD. We performed a systematic review of the clinical and radiological features and outcomes of patients who underwent surgery for degenerative spine problems with a subsequent diagnosis of spine CPPD.

Methods

This systematic review was conducted in accordance with the Preferred Reporting Items for Systematic Reviews and Meta-Analyses (PRISMA) guidelines.

Results

Two hundred and eleven articles from electronic databases met our search criteria for the systematic review. Thirty-seven clinical studies comprising 153 patients aged 23 to 83 years were included. Of these, 68 were male, and 85 were female. A total of 33.7% involved the cervical region, 16.2% involved the thoracic region, and 50% involved the lumbar region. The common comorbidities included hypertension, dyslipidemia, diabetes mellitus (DM), and cardiac and renal issues. The common radiological features included nonspecific degenerative changes, an epidural mass, and calcified masses, among others. The most common intraoperative finding was chalky white deposits. The vast majority of patients had good postoperative outcomes. Our institutional case series included six patients. These were analyzed and reported separately in the text.

Conclusions

Spinal CPPD is likely much more common than we currently report, primarily because it is underdiagnosed or misdiagnosed for other pathologies. This review investigated the epidemiology, presenting features, imaging, histopathological, and intraoperative findings of this disease. Future prospective studies should focus on establishing standardized diagnostic criteria combining preoperative imaging characteristics with intraoperative findings to improve recognition of spinal CPPD. Additionally, investigations into optimal medical management strategies for patients with concurrent CPPD and degenerative spine disease who may not require surgical intervention would be valuable.

## Introduction

Calcium pyrophosphate deposition disease (CPPD), also known as pseudogout, is a crystal-induced inflammatory arthropathy that commonly affects the elderly population [1,2]. Its clinical presentation ranges from an asymptomatic course to acute or chronic inflammatory arthritis [3]. It frequently involves large joints such as the hips, knees, shoulders, wrists, and ankles, while occurrence in the spine is considered rare [4]. In addition to aging as a risk factor, CPPD might also be a monogenic familial disease or be caused by various metabolic disorders, such as hereditary hemochromatosis, primary hyperparathyroidism, and hypomagnesemia [5]. An association has also been shown with the use of loop diuretics and possibly osteoarthritis [6]. While the pathophysiology is well-understood, the clinical identification of the disease remains a significant challenge, particularly when it presents in non-appendicular sites [7]. Consequently, histopathological confirmation remains the gold standard for definitive diagnosis [8]. Despite scattered case reports, no prior review has systematically summarized the clinical, imaging, and intraoperative characteristics of spinal CPPD across all spinal regions.

CPPD is thought to affect 4-7% of adults in the United States (US) [9]. There is, however, a relative paucity of data on spinal involvement in CPPD. One study reported spinal involvement in 24.3% of patients with known CPPD [10]. This was, however, in a series of 37 patients in an academic rheumatology center. We are not aware of any other works that have reported a similar incidence. Considering that magnetic resonance imaging (MRI) findings usually reveal adjacent soft tissue enhancement with increased signal intensity and extensive erosion changes, many patients at initial presentation are managed as osteomyelitis or discitis [4]. It has also been reported to present as skip lesions mimicking infection after lumbar fusion [9]. The obvious implication of this is that there is a potential diagnostic dilemma with subsequent mismanagement or even avoidable surgery if CPPD is mistaken for spinal degenerative changes or another alternate diagnosis.

This study has two primary objectives: to report an institutional case series of six patients who underwent spinal surgery for degenerative disease between 2019 and 2023 and had histopathologically confirmed CPPD as an incidental intraoperative finding; and to perform a systematic review of published literature to characterize the clinical, radiological, intraoperative, and histopathological features of spinal CPPD, as well as surgical outcomes in this population. Through synthesis of our institutional experience with the existing literature, we aim to improve recognition of this underdiagnosed condition and provide evidence regarding its impact on surgical prognosis.

## Materials and methods

Case series collection

The study was conducted at The Walton Centre NHS Foundation Trust, Liverpool. We reviewed the medical records from our tertiary neurosurgery center from 2019 to 2023 to identify consecutive patients who underwent spinal surgery for degenerative spinal conditions and whose collected operative samples confirmed the presence of CPPD. We summarized the clinical presentation, radiological and operative findings, and postoperative outcomes of the six identified cases.

All operative specimens were sent fresh to the pathology department for routine processing. Tissue samples were fixed in 10% formalin, embedded in paraffin, sectioned at 4-5 μm thickness, and stained with hematoxylin and eosin (H&E). When gritty or chalky material was encountered intraoperatively and noted by the surgeon, or when crystalline deposits were suspected on initial H&E examination, additional unstained sections were prepared for examination under polarized light microscopy. The diagnosis of CPPD was confirmed by demonstration of rhomboid or rod-shaped crystals showing weakly positive birefringence under polarized light, along with characteristic basophilic aggregates in surrounding tissue.

Systematic review methodology

This systematic review was conducted in accordance with the Preferred Reporting Items for Systematic Reviews and Meta-Analyses (PRISMA) guidelines [11]. The search for articles was performed in Ovid Medical Literature Analysis and Retrieval System Online (MEDLINE), Embase, the Cochrane Central Register of Controlled Trials (CENTRAL), and the Cochrane Database of Systematic Reviews (CDSR) (Appendices 1-5).

We used the keywords ("Chondrocalcinosis" OR "CPPD" OR "Calcium Pyrophosphate Dihydrate Disease" OR "Pseudogout") AND ("Spine" OR "Spinal Vertebra") AND ("Laminectomy" OR "Surgical Procedure"). The reference lists of the retained papers were also screened manually to search for any other studies that might be eligible for the research.

Study selection criteria and data extraction

Inclusion Criteria

The inclusion criteria encompassed a range of study designs, including case reports, case series, prospective observational studies, and retrospective cohort studies that reported original patient data. Only studies published in the English language were considered. The review was limited to studies published within the last 20 years (January 2004 to June 2024). This timeframe was selected for two reasons: modern high-resolution MRI techniques that can better characterize spinal pathology have evolved significantly since the early 2000s, with older imaging potentially using different diagnostic thresholds; and diagnostic criteria for CPPD, including standardized histopathological assessment with polarized light microscopy, became more widely adopted in the 2000s. The population of interest included adult patients (aged 18 years or older) who underwent spinal surgery for degenerative spine conditions. Eligible interventions comprised any surgical procedures for degenerative spine disease, including but not limited to laminectomy, laminoplasty, spinal fusion or instrumentation, decompression procedures, facet joint cyst excision, and epidural mass resection. The primary outcome was the histopathological confirmation of CPPD in intraoperative tissue samples using polarized light microscopy, demonstrating characteristic weakly positive birefringent rhomboid or rod-shaped crystals. Additionally, studies were required to provide sufficient clinical data to allow extraction of at least three of the following variables: patient demographics, clinical presentation, radiological findings, surgical procedure performed, intraoperative findings, histopathological confirmation, or clinical outcomes.

Exclusion Criteria

Studies meeting any of the following criteria were excluded: study types such as systematic reviews and meta-analyses, conference abstracts without full-text publications, letters to the editor, editorials, and commentaries lacking original patient data; studies published in languages other than English; and studies published before January 2004. Additionally, studies involving pediatric populations, as well as animal, cadaveric, biomechanical, or in vitro studies, were excluded. Studies were also excluded if they involved spinal CPPD managed conservatively without surgical intervention or focused exclusively on CPPD at non-spinal sites. Reports in which CPPD was suspected but not histopathologically confirmed were not considered eligible. Finally, duplicate publications reporting the same patient cohort and studies with insufficient extractable data were excluded.

All papers identified through the search strategy were independently reviewed by two authors (OA and MI) to assess their suitability based on the predetermined inclusion and exclusion criteria. Disagreements between reviewers were resolved through discussion, with consultation of a third reviewer (NS) when consensus could not be reached. To partially address the limitation of the 20-year cutoff, we manually screened reference lists of all included papers and relevant reviews.

A standardized data extraction template was created and piloted on five randomly selected studies. Data extracted included study characteristics, patient demographics, spinal region involved, clinical presentation, medical comorbidities, radiological findings, surgical procedure(s) performed, intraoperative findings, histopathological findings, and clinical outcomes. Data were extracted independently by two reviewers.

## Results

Case series

We present a case series of six patients who underwent spinal surgery for degenerative conditions at our tertiary neurosurgery center between 2019 and 2023. In all cases, intraoperative samples confirmed the presence of CPPD, which was an incidental finding that led us to investigate this pathology further. The detailed characteristics of each case are presented in Table 1, and comprehensive case narratives follow below.

**Table 1:** Our institutional case series The table above represents our series of six patients who had surgery for degenerative spine conditions, and in whom intraoperative samples confirmed the presence of CPPD. As we systematically reviewed our histopathological data, the frequency of this finding became apparent, leading us to conduct this broader systematic review of the literature. AF: atrial fibrillation; LBP: lower back pain; Hx: history; CPPD: calcium pyrophosphate deposition disease; DM: diabetes mellitus

Age	Sex	Presenting Complaints	Past Medical History	Radiological Findings	Surgery	Outcome
72	F	Right-sided sciatica x 3/12	Paroxysmal AF large cystic adenoma under surveillance	Right L5/S1 cyst	Right L5/S1 fenestration/excision of facet joint cyst	Improved post op and off usual analgesics
84	F	6/52 hx of LBP + bilateral sciatica and episodes of urinary incontinence	Hypertension; previous thyroidectomy; previous cardiac stent placement for angina; on clopidogrel	Posterior L1-3 epidural mass compressing the thecal sac	Posterior lumbar decompression/exploration	Good outcome; mobilizing independently by the 8^th^ day post op with very little analgesia
78	F	2-year hx of worsening numbness in arms and fingers and heaviness in legs following a fall	Ulcerative colitis; type 2 DM; arthritis; asthma; previous right hip replacement	C3/4 stenosis and C3/4 anterolisthesis	C3/4 laminectomy	Myelopathic features had improved by 3/12 follow-up
83	F	Back pain and bilateral leg heaviness	Hypothyroidism	Multilevel degenerative changes with significant L3/4 stenosis	L3/4 decompression	Significantly improved symptoms postoperatively
64	M	3/12 hx of bilateral leg pain, worse on the right side. Associated saddle anesthesia and feeling of incomplete bladder emptying	Previous L5-S1 posterolateral fusion following L5 fracture	T1/L1 facet joint cyst compressing the conus	T12/L1 conus decompression	Discharged day after surgery with resolved b/l sciatica and a normal post-void bladder scan
78	M	Bilateral claudicant symptoms	Prostatism; previous bilateral inguinal hernia repair; colonic polyps	Multilevel degenerative spine with L4/5 anterolisthesis, facetal hypertrophy, and lateral recess narrowing	L4/5 decompression	Discharged the day after surgery with improved symptoms

Case 1: 72-Year-Old Female With Right L5/S1 Facet Joint Cyst

Clinical presentation: A 72-year-old female presented to our clinic with a three-month history of right-sided sciatica. Her past medical history was significant for paroxysmal atrial fibrillation, for which she was on appropriate medical management, and a large cystic adenoma under surveillance by her endocrinology team.

**Figure 1:**
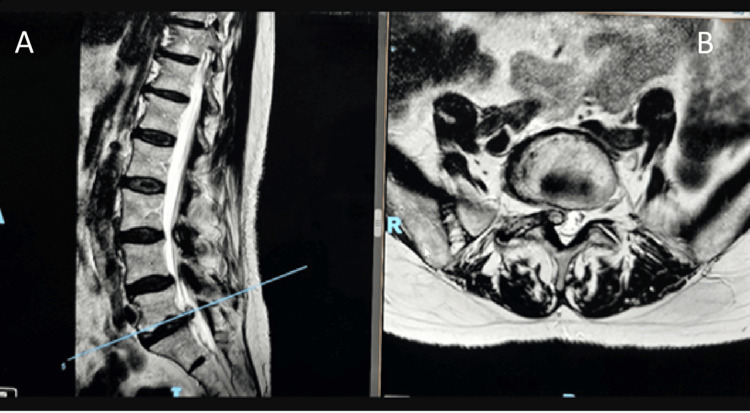
MRI lumbosacral spine Shows a representative MRI lumbar spine from one of our cases, demonstrating at the L5-S1 level a large right paracentral disc extrusion compressing the thecal sac, occupying and effacing the right lateral recess with compression to the transiting nerve root. There is also an associated facet joint cyst. (A) sagittal view; (B) axial view

Radiological findings: MRI of the lumbosacral spine revealed a right-sided facet joint cyst at the L5/S1 level (Figure 1). The cyst was causing significant compression of the nerve root, correlating with her clinical presentation of right-sided sciatica.

**Figure 2:**
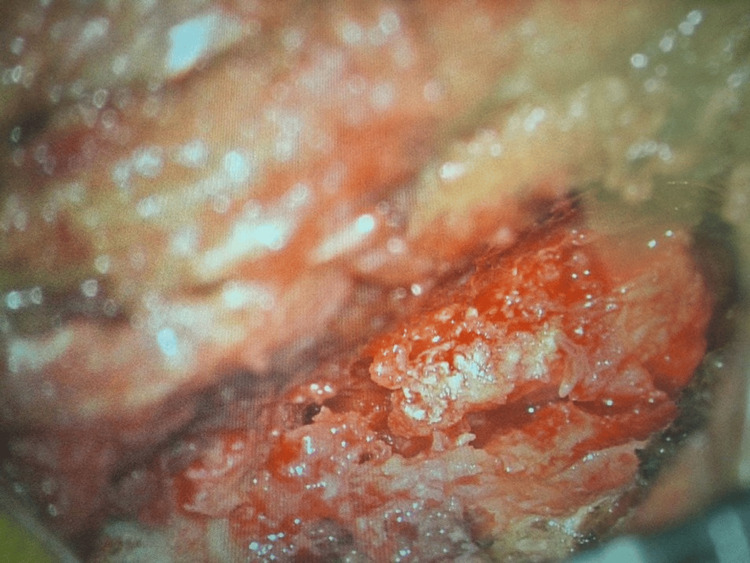
Gross intraoperative findings Demonstrates the characteristic intraoperative findings of gritty or white chalky material interspersed within the hypertrophied ligaments, which is pathognomonic for CPPD. CPPD: calcium pyrophosphate deposition disease

Surgical intervention: The patient underwent a right L5/S1 fenestration with excision of the facet joint cyst. During the procedure, we encountered characteristic findings of CPPD: gritty, white, chalky material interspersed within the hypertrophied ligaments (Figure 2). Intraoperative samples were sent for histopathological examination.

**Figure 3:**
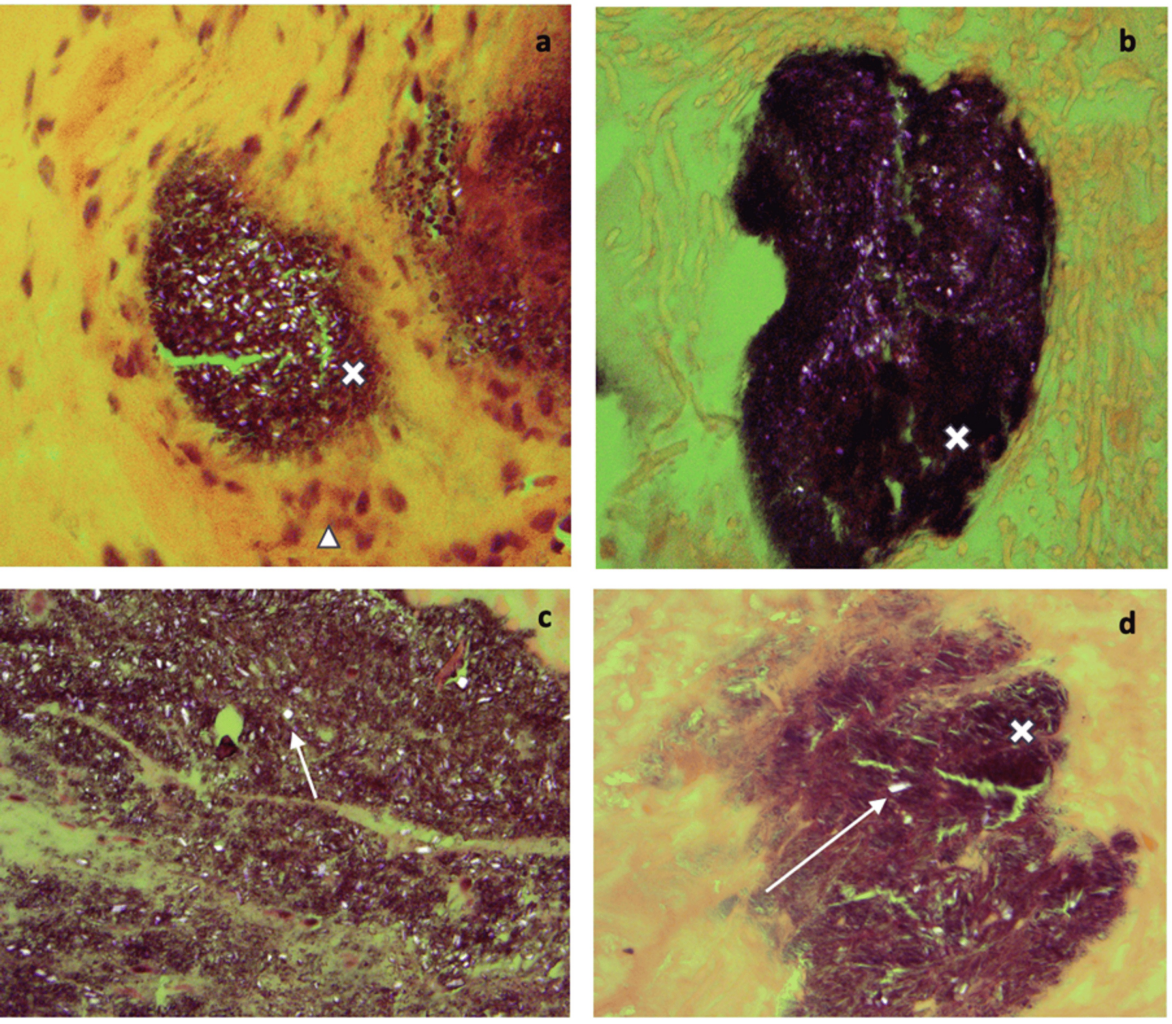
H&E staining under polarized light Routine H&E staining of calcium pyrophosphate crystal deposition disease under polarized light. Distinct basophilic aggregates (x) contain rhomboid- to rod-shaped crystals (arrow) that demonstrate positive birefringence under polarized light. Focal chronic inflammation is seen in the form of histiocytes (arrowhead). Specific pathological features being highlighted are (a) shows a basophilic aggregate (the dark purple/brown area marked with 'x') with crystalline material demonstrating birefringence (the sparkling/bright areas within); (b) shows a larger aggregate under polarized light, demonstrating the characteristic appearance of CPPD crystals en masse; (c) shows crystals dispersed within tissue (marked with an arrow), demonstrating the rod-shaped to rhomboid morphology with positive birefringence; and (d) shows focal chronic inflammation with histiocytes (inflammatory cells) surrounding the crystalline deposits (marked with an arrow). CPPD: calcium pyrophosphate deposition disease

Histopathological findings: Routine H&E staining under polarized light demonstrated distinct basophilic aggregates containing rhomboid to rod-shaped crystals that showed positive birefringence, confirming the diagnosis of CPPD (Figure 3). Focal chronic inflammation was noted in the form of histiocytes.

Outcome: The patient experienced significant improvement in her sciatica postoperatively and was able to discontinue her regular analgesics. At follow-up, she remained symptom-free and satisfied with the outcome.

Case 2: 84-Year-Old Female With Posterior Lumbar Epidural Mass

Clinical presentation: An 84-year-old female presented with a six-week history of lower back pain associated with bilateral sciatica and episodes of urinary incontinence. Her medical history included hypertension managed with antihypertensive medications, a previous thyroidectomy, and a cardiac stent placement for angina, for which she was maintained on clopidogrel.

Radiological findings: MRI of the lumbar spine revealed a posterior epidural mass extending from L1 to L3, causing significant compression of the thecal sac. Given the clinical presentation and imaging findings, there was initial concern about the nature of the mass, with differential diagnoses including infection, tumor, or degenerative pathology.

Surgical intervention: The patient underwent posterior lumbar decompression and exploration of the epidural space. Intraoperatively, we identified characteristic chalky white deposits within the hypertrophied ligamentum flavum. The deposits had a gritty consistency and were carefully removed along with the decompression.

Histopathological findings: Histopathological examination of the operative specimens confirmed the presence of calcium pyrophosphate crystal deposits, with the characteristic rhomboid crystals visible under polarized light microscopy.

Outcome: The patient had an excellent postoperative course. She was mobilizing independently by the eighth postoperative day with minimal analgesia requirements. Her bladder symptoms resolved completely, and she reported significant improvement in her back pain and radicular symptoms at her follow-up appointments.

Case 3: 78-Year-Old Female With Cervical Myelopathy

Clinical presentation: A 78-year-old female presented with a two-year history of progressively worsening numbness in her arms and fingers, along with a sensation of heaviness in her legs. These symptoms developed following a mechanical fall. Her past medical history was notable for ulcerative colitis managed with immunosuppressive therapy, type 2 diabetes mellitus, arthritis affecting multiple joints, asthma, and a previous right hip replacement.

Radiological findings: Cervical spine MRI demonstrated significant spinal canal stenosis at the C3/4 level, with associated C3/4 anterolisthesis. The stenosis was causing cord compression, which correlated with her myelopathic symptoms.

Surgical intervention: The patient underwent a C3/4 laminectomy for decompression of the cervical spinal cord. During the laminectomy, we encountered calcified ligamentum flavum with associated whitish, gritty material consistent with CPPD deposition.

Histopathological findings: The operative specimens were sent for histopathological analysis, which confirmed extensive CPPD within the ligamentum flavum. The characteristic birefringent crystals were clearly visible on polarized light microscopy.

Outcome: At her three-month follow-up appointment, the patient reported significant improvement in her myelopathic features. The numbness in her upper extremities had decreased considerably, and the heaviness in her legs had improved, allowing for better mobility and independence.

Case 4: 83-Year-Old Female With Lumbar Spinal Stenosis

Clinical presentation: An 83-year-old female with a background of hypothyroidism presented with back pain and bilateral leg heaviness. Her symptoms were consistent with neurogenic claudication secondary to lumbar spinal stenosis.

Radiological findings: MRI of the lumbar spine showed multilevel degenerative changes with the most significant stenosis at the L3/4 level. The imaging revealed hypertrophied facet joints and ligamentum flavum contributing to the central canal narrowing.

Surgical intervention: The patient underwent L3/4 decompression surgery. During the procedure, we noted white chalky material within the hypertrophied ligamentum flavum, which prompted us to send specimens for histopathological examination.

Histopathological findings: Pathological analysis confirmed the presence of CPPD within the excised ligamentum flavum, demonstrating the characteristic crystal deposition pattern seen with this condition.

Outcome: Postoperatively, the patient experienced significant improvement in her symptoms. Her back pain decreased substantially, and the heaviness in her legs resolved, allowing her to ambulate more comfortably. She was very satisfied with the surgical outcome.

Case 5: 64-Year-Old Male With Conus Compression

Clinical presentation: A 64-year-old male presented with a three-month history of bilateral leg pain, which was more severe on the right side. He also described saddle anesthesia and a sensation of incomplete bladder emptying, raising concern for cauda equina syndrome. His past surgical history included L5-S1 posterolateral fusion following an L5 fracture.

Radiological findings: MRI of the thoracolumbar spine revealed a T12/L1 facet joint cyst causing compression of the conus medullaris. Given his history of previous spinal instrumentation at a distant level, this represented a new pathology developing superior to his previous fusion.

Surgical intervention: The patient underwent urgent T12/L1 decompression surgery to relieve the compression on the conus. Intraoperatively, we discovered a hemorrhagic cyst associated with extensive CPPD deposition. The cyst was excised, and the conus was adequately decompressed.

Histopathological findings: The resected cyst wall and surrounding tissue demonstrated significant calcium pyrophosphate crystal deposition. The hemorrhagic nature of the cyst with concurrent CPPD was an unusual but well-documented finding.

Outcome: The patient had an excellent recovery. He was discharged the day after surgery with complete resolution of his bilateral sciatica. His saddle anesthesia resolved, and a post-void bladder scan demonstrated normal bladder function. At follow-up, he remained symptom-free.

Case 6: 78-Year-Old Male With Neurogenic Claudication

Clinical presentation: A 78-year-old male presented with bilateral claudicant symptoms affecting his lower limbs. His medical history included prostatism, previous bilateral inguinal hernia repairs, and colonic polyps that were being monitored with regular colonoscopy.

Radiological findings: Lumbar spine MRI demonstrated multilevel degenerative spine disease with the most significant pathology at L4/5. There was grade 1 anterolisthesis of L4 on L5, facetal hypertrophy, and lateral recess narrowing, all contributing to his claudication symptoms.

Surgical intervention: The patient underwent L4/5 decompression surgery. During the procedure, we encountered an extradural mass that was firmly attached to the dura. Upon further dissection, we identified whitish and gritty connective tissue consistent with CPPD deposition. The tissue was carefully dissected from the dura, and adequate decompression was achieved.

Histopathological findings: Histological examination confirmed extensive calcium pyrophosphate crystal deposition within the hypertrophied ligamentum flavum and epidural tissue. The crystals demonstrated the typical positive birefringence under polarized light.

Outcome: The patient had an uncomplicated postoperative course and was discharged the day following surgery with significant improvement in his claudication symptoms. At his outpatient follow-up, he reported excellent pain relief and improved walking tolerance.

Summary of case series findings

Across our institutional case series of six patients, several patterns emerged. The mean age was 76 years (range 64-84 years), with four females and two males. Four cases involved the lumbar spine, and two involved the cervical spine. None of our patients had a pre-existing diagnosis of CPPD in other joints, highlighting that spinal CPPD may occur as an isolated finding. The most common intraoperative finding was white chalky or gritty material within hypertrophied ligaments, which served as a characteristic macroscopic indicator of CPPD deposition (Figure 2).

All specimens demonstrated the pathognomonic histological features of CPPD when examined under polarized light microscopy (Figure 3): rhomboid to rod-shaped crystals with positive birefringence, distinct basophilic aggregates, and associated chronic inflammation. Importantly, all six patients had favorable postoperative outcomes, with five patients experiencing significant symptom improvement and one patient (Case 5) achieving complete resolution of his presenting symptoms. This finding is consistent with the established literature showing that the presence of CPPD does not appear to negatively impact surgical outcomes in patients with degenerative spine disease.

The discovery of CPPD in these six consecutive surgical cases was initially serendipitous. However, as we began systematically collecting and analyzing specimens, it became apparent that spinal CPPD might be more common than previously recognized, likely due to underdiagnosis or misattribution to other degenerative processes. This observation prompted us to conduct the systematic review presented in the Discussion section to better understand the prevalence, presentation, and clinical significance of this condition.

## Discussion

This study aims to report our institutional case series of surgically confirmed spinal CPPD; systematically review the published literature on spinal CPPD presentation, imaging, and surgical outcomes; and characterize the clinical and radiological features that may aid in preoperative recognition. These findings may inform future prospective studies to establish the true prevalence of spinal CPPD and identify patients who might benefit from targeted medical therapy rather than surgery.

Systematic review results

Study Results and Flowchart

**Figure 4:**
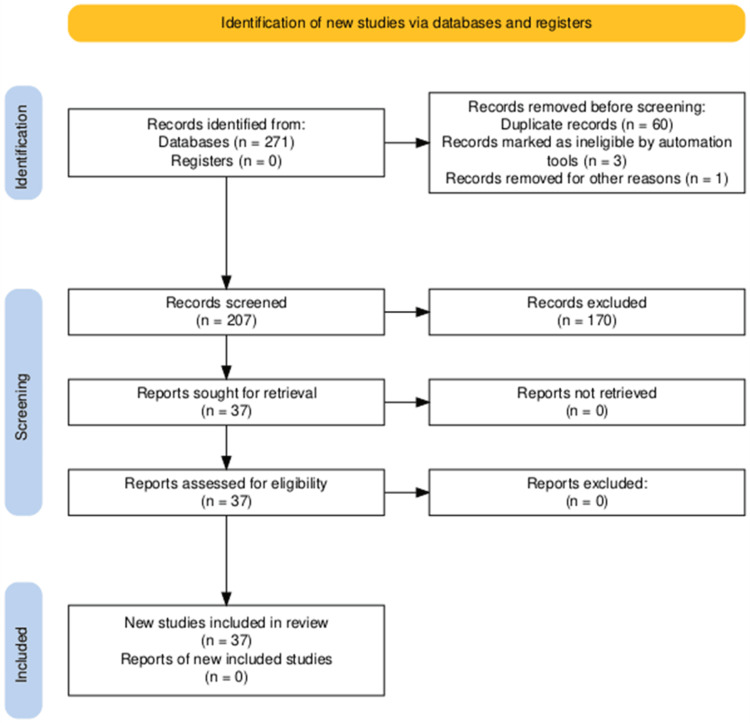
PRISMA flowchart PRISMA: Preferred Reporting Items for Systematic Reviews and Meta-Analyses

After screening out duplicates, a total of 211 papers met our search criteria through the search of relevant electronic databases, including Ovid MEDLINE, Embase, CENTRAL, and CDSR. Upon screening the titles and abstracts and assessing the quality of the papers, we identified 37 papers that were ultimately included in the study. The systematic review process is illustrated in Figure 4. Among the included studies, 30 were case reports, two were retrospective cohort studies [12,13], and five were case series. Notable among the larger studies was a pathologic examination of 77 surgical cases examining anatomical locations and correlates of calcium pyrophosphate crystal deposits of the spine [12], and a study on the role of CPPD deposition in the postoperative outcome of lumbar spinal stenosis patients [13].

Patient Characteristics

The ages ranged between 23 and 83 years. The average age was 66 years. The total number of patients included in the study was 153. Of these, 68 were male, and 85 were female. In terms of the part of the spine involved, 33.7% involved the cervical region, 16.2% involved the thoracic region, and 50.1% involved the lumbar region. The comorbidities, such as hypertension, dyslipidemia, diabetes mellitus (DM), and other cardiac and renal issues, varied with the occurrence of common chronic illnesses in this age group of patients. Notably, only four of the patients in this series had a diagnosis of extraspinal gout, and none mentioned pseudogout as a comorbidity. Virtually all the patients had satisfactory outcomes in terms of improvement or non-progression of their symptoms. However, three patients died within a few months of surgery, which was related to underlying medical conditions. One notable case report described disorders of tumoral calcification of the spine, including a patient with severe renal impairment who opted to discontinue dialysis. The available radiological features, clinical history at presentation, and gross intraoperative findings are detailed in Tables 2-4. The full clinical characteristics, radiological findings, and surgical outcomes of the included studies are summarized in Table 5 [12-48].

**Table 2:** Gross intraoperative findings NOS: not otherwise specified

Findings	Number (%)
“Cyst”	6 (9.23%)
“Calcified ligamentum flavum”	4 (6.15%)
“Calcified mass”	6 (9.23%)
“Mass lesion (NOS)”	2 (3.08%)
“Chalky white deposit”	44 (67.69%)
“Gritty material”	1 (1.54%)
“Glistening granular material”	2 (3.08%)

**Table 3:** Clinical history at presentation The table above explores the clinical presentation of each patient. As might be expected, many patients had more than one of the above presenting complaints.

Presentation	Number (%)
Pain	33 (21.7%)
Previous instrumentation	4 (2.63%)
Previous trauma	2 (1.32%)
Limb weakness	6 (3.95%)
Myelopathy	24 (15.79%)
Cauda equina syndrome (CES)	4 (2.63%)
Fever	1 (0.66%)
Neck swelling	1 (0.66%)
Unavailable	77 (50.66%)

**Table 4:** Radiological features in number and corresponding percentage

Radiological Features	Number (%)
Degenerative changes	30 (42.86%)
Cyst	7 (10%)
Epidural mass (not otherwise specified)	13 (18.57%)
Vertebral body infiltrative mass	1 (1.43%)
Calcified mass	11 (15.71%)
Imaging findings, highly suggestive of calcium pyrophosphate deposition disease (CPPD)	6 (8.57%)
Calcified ligamentum flavum	1 (1.43%)
Ossified posterior longitudinal ligament (OPLL)	1 (1.43%)

**Table 5:** Complete dataset of all studies used in the systematic review TKR: total knee replacement; HTN: hypertension; DM: diabetes mellitus; CPPD: calcium pyrophosphate deposition disease; CABG: coronary artery bypass grafting; OPLL: ossified posterior longitudinal ligament; COPD: chronic obstructive pulmonary disease; CAD: coronary artery disease; CHF: congestive heart failure; Ca: cancer; NA: not available; LF: ligamentum flavum; OA: osteoarthritis; ODI: Oswestry Disability Index; CES: cauda equina syndrome

Author/Publication Year	Ref	Type of Study	Age	Sex	Part of Spine	Clinical Features	Comorbidities	Radiographic Findings	Surgical Procedure	Intraoperative Findings	Outcome
Dau J, et al. (2021)	[12]	Retrospective Cohort	Mean 71.3 yrs (range 43-90)	36M, 41F (Total 77)	Cervical (26), Thoracic (6), Lumbar (45)	Myelopathy; radiculopathy; neurogenic claudication	NA	Calcification of ligamentum flavum, discs, and posterior atlantoaxial membrane	Decompression and/or fusion (various)	Presence of CPPD crystals in 77/77 surgical specimens	Generally improved; focused on histological correlation
Ariyawatkul T, et al. (2019)	[13]	Retrospective Cohort	Mean 67.5 yrs	12M, 11F (Total 23)	Lumbar	Neurogenic claudication; chronic back pain	HTN; DM; OA; Obesity	Lumbar spinal stenosis with facet and LF calcification	Lumbar decompression (laminectomy)	CPPD deposits within the ligamentum flavum	Significant improvement in ODI scores postoperatively
Hallstead SJ, et al. (2025)	[14]	Case Series	63-85 yrs	5M 5F	Lumbar (9), Thoracic (1)	90% chronic, 10% acute pain; 4 with prior instrumentation; 1 with prior trauma	NA	Central canal stenosis (8); neuroforaminal narrowing (5); septic arthropathy (4); synovial cyst (3); degeneration (2); epidural mass/abscess (2)	NA	Spinal cyst	2 treated for CPPD; 2 prescribed antibiotics; 2 poor outcomes; 1 had CPPD in wrist aspirate 4 years later
Choi SJ, et al. (2024)	[15]	Case report	58, 64	M, F	Cervical	Myelopathy (both)	NA	C3/4 and C5/6 CPPD calcification in ligamentum flavum	Full endoscopic cervical laminotomy (both cases)	CPPD in ligamentum flavum	Improved (both)
Afzal S, et al. (2023)	[16]	Case report	65	F	Cervical	Progressive bilateral upper limb weakness and gait disturbance (myelopathy)	NA	C5/6 canal stenosis with cord signal change	C5/6 laminectomy	Calcified ligamentum flavum - CPPD	Improved
Pongmanee S, et al. (2021)	[17]	Case report	77	F	Cervical	Neck pain, bilateral hand numbness, bilateral leg weakness (myelopathy)	Hypertension; dyslipidemia	Retro-odontoid pseudotumor; multilevel cervical spondylosis	C1-C3 laminectomy with posterior fixation	Chalky white deposit	Improved
Lo PC, et al. (2021)	[18]	Case report	70	M	Lumbar	Low back pain and right-sided radiculopathy	Hypertension; DM	Extradural mass at L3/4	L3/4 laminectomy and excision of extradural mass	Chalky white deposit	Improved
Liao JH, et al. (2021)	[19]	Case report	64	M	Cervical	Neck pain; bilateral upper and lower limb numbness and weakness; myelopathic gait	DM; hypertension; dyslipidemia	C2/3 posterior epidural mass with cord compression	C1-C3 laminectomy	Chalky white deposit (CPPD confirmed)	Improved
Gewolb DP, et al. (2021)	[20]	Case report	68	M	Lumbar	Back pain; bilateral leg weakness; bowel and bladder dysfunction (CES)	HTN; DM; CAD; CHF	L1 intradural/extramedullary mass	L1 laminectomy; intradural exploration; mass resection	Intradural calcified mass - CPPD	Improved
Chakravorty A, et al. (2021)	[21]	Case report	78	F	Lumbar	Back pain, bilateral leg weakness, cauda equina syndrome	HTN; COPD	Posterior epidural mass at L1; intradural extension	L1 laminectomy; intradural mass excision	Intradural CPPD deposit	Improved
Miura K, et al. (2020)	[22]	Case report	64	M	Thoracic	Progressive gait disturbance; bilateral leg weakness (myelopathy)	NA	T10/11 calcification of ligamentum flavum with cord compression	T10/11 laminectomy	Calcified ligamentum flavum - CPPD	Improved
Chang DG, et al. (2020)	[23]	Case report	71, 79	M, F	Cervical	Neck pain; myelopathy (both)	1: HTN; 2: DM, HTN	1: C4/5 CPPD + crowned dens; 2: C4/5 CPPD + crowned dens	1: C3-C5 laminoplasty; 2: C3-C5 laminoplasty	Chalky white deposit	Improved (both)
Shen G, et al. (2019)	[24]	Case report	67	M	Thoracic	Back pain; bilateral leg weakness	Hypertension	Tophaceous pseudogout at multiple thoracic levels on 18F-FDG PET/CT	Thoracic decompression	Chalky white deposits	Improved
Madhavan K, et al. (2018)	[25]	Case report	49, 72, 84	2F 1M	Cervical	Neck pain; myelopathy	1: HTN; 2: NA; 3: NA	Retro-odontoid mass (all 3)	Transdural resection of retro-odontoid cysts (all 3)	Cyst with CPPD	Improved (all 3)
Ng IBY, et al. (2016)	[26]	Case report	70	M	Cervical	Neck pain; myelopathy	Hypertension	Posterior C1-C2 mass with cord compression	C1-C2 laminectomy	Chalky white deposit - CPPD	Improved
Morino T, et al. (2016)	[27]	Case report	39	M	Cervical	Neck pain (acute); tetraparesis (on follow-up)	Coffin-Lowry syndrome	CPPD calcification at C5/6 yellow ligament (initial); progression at C3/4 and C4/5 (8-yr follow-up)	C5/6 laminectomy (initial); repeat C3/4 and C4/5 decompression (follow-up)	Calcified ligamentum flavum - CPPD	Improved; stable at 8-year follow-up
Kobayashi T, et al. (2016)	[28]	Case report	59	M	Cervical	Acute neck pain; limited range of motion	Hypertension	Calcification in the cervical yellow ligament at C3/4	Laminectomy C3/4	Calcified ligamentum flavum - CPPD	Improved
Mori K, et al. (2015)	[29]	Case report	79	F	Cervical	Neck pain; myelopathy; gait disturbance	Generalized CPPD	Calcification of the posterior atlantoaxial membrane	C1 laminectomy and decompression	Calcified mass - CPPD	Improved
Leung J, et al. (2015)	[30]	Case report	72	M	Cervical	Cervical myelopathy	NA	C3-C5 epidural mass mimicking an abscess.	NA	NA	Improved
Luksanapruksa P, et al. (2013)	[31]	Case report	74	F	Cervical	Neck pain; progressive myelopathy	Hypertension	Retro-odontoid CPPD mass with cord compression	Transoral resection of retro-odontoid mass	Chalky white deposit - CPPD	Improved
Cacciotti G, et al. (2013)	[32]	Case report	56	M	Lumbar (filum terminale)	Low back pain; bilateral leg pain	NA	Calcified mass at filum terminale	Excision of filum terminale mass	CPPD in filum terminale	Improved
Srinivasan V, et al. (2012)	[33]	Case report	55	M	Thoracic	Back pain; progressive paraparesis	Gout	Tophaceous deposit at T8/9	T8/9 laminectomy	Chalky white tophaceous deposit - CPPD	Improved
Schmeer JL and Cruz ES (2012)	[34]	Case report	61	F	Cervical	Acute cervical myelopathy; upper and lower limb weakness	NA	C5-6 ligamentum flavum calcification	NA	NA	Improved
Odate S, et al. (2012)	[35]	Case report	79	F	Thoracic	Progressive thoracic myelopathy after lumbopelvic fusion	Previous lumbopelvic fusion	Proximal junctional vertebral compression fracture with CPPD at T9	T9 laminectomy	Chalky white deposit - CPPD	Improved
Namazie MR and Fosbender MR (2012)	[36]	Case report	74	M	Lumbar	Low back pain; bilateral leg pain	DM; HTN	CPPD in multiple lumbar facet joints (L3/4, L4/5, L5/S1)	Lumbar facet joint decompression	Chalky white deposit - CPPD	Improved
Mavrogenis AF, et al. (2010)	[37]	Case report	74	F	Cervical	Neck pain; progressive myelopathy	NA	C3/4 calcified mass compressing the spinal cord	C3/4 laminectomy	Chalky white deposit - CPPD	Improved
Brent A and Hartley R (2010)	[38]	Case report	77	M	Cervical	Recurrent falls; myelopathy	Hypertension	Retro-odontoid CPPD mass	Transoral decompression	Chalky white deposit - CPPD	Improved
Sethi KS, et al. (2007)	[39]	Case report	73	F	Cervical	Progressive myelopathy; cervicomedullary compression	Hypertension	Massive C1-C2 CPPD deposit with vertebral artery encasement and intradural extension	C1-C2 laminectomy; intradural exploration	Chalky white deposit - CPPD; intradural extension	Improved
Scavarda D, et al. (2007)	[40]	Case report	60	F	Cervical	Neck pain; myelopathy	Previous suboccipital craniotomy 28 yrs prior	Large retroodontoid CPPD mass at C1-C2	Transoral + posterior decompression	Chalky white tumoral deposit - CPPD	Improved
Lam HY, et al. (2007)	[41]	Case series	23-75 yrs	2M 2F	Lumbar (4 cases)	Low back pain; radiculopathy (all 4)	1: none; 2: none; 3: gout; 4: none	CPPD deposits in lumbar facet joints (all 4 cases)	Lumbar decompression (all 4)	Chalky white deposit - CPPD (all 4)	Improved (all 4)
Giulioni M, et al. (2007)	[42]	Case report	72	M	Thoracic	Progressive myelopathy; lower limb weakness	NA	Calcified ligamentum flavum at T4/5	T4/5 laminectomy	Calcified ligamentum flavum - CPPD	Improved
Doita M, et al. (2007)	[43]	Case report	67	M	Cervical	Neck pain; progressive myelopathy	NA	CPPD in the transverse ligament of the atlas; retro-odontoid mass	Transoral decompression	Chalky white deposit - CPPD	Improved
Carlson AP, et al. (2007)	[44]	Case report	66	F	Lumbar	Back pain, radiculopathy, cauda equina syndrome	NA	Tumoral calcification at L5/S1	L5/S1 laminectomy and mass excision	Chalky white deposit - CPPD	Improved
Lin SH, et al. (2006)	[45]	Case report	64	F	Cervical	Neck pain; myelopathy	Gout	CPPD in ligamentum flavum and retro-odontoid mass	C1-C3 laminectomy; transoral decompression	Chalky white deposit - CPPD	Improved
Paolini S, et al. (2005)	[46]	Case report	36, 43	F, F	Thoracic (both)	Left-sided chest pain (both)	1: NA; 2: NA	1: T9/10 lateral disc herniation; 2: T9/10 nodular lesion	1: T9 hemilaminectomy; 2: T9 hemilaminectomy	1: Glistening, finely granular material; 2: Soft, degenerated material	Improved (both)
Nakase H, et al. (2005)	[47]	Case report	68	F	Cervical	Left shoulder pain; tetraparesis (myelopathy)	NA	C4/5 calcified mass	Left C4/5 hemilaminectomy	CPPD confirmed histologically	Improved
Griesdale DE Jr, et al. (2004)	[48]	Case report	70	F	Cervical	Myelopathy	NA	Retroodontoid mass - CPPD in the transverse atlantal ligament	Transoral resection	CPPD in the transverse atlantal ligament	Improved

Literature review findings

This finding is consistent with the established role of conservative care in the management of spinal degenerative diseases [49-52]. In this review, 30 of the 37 studies included for analysis were case reports, whereas five were case series. This finding highlights the relatively uncommon nature of spinal CPPD/pseudogout. Other researchers have shown that not only is axial CPPD rare, but it is also underdiagnosed [53,54]. The symptoms and known radiological findings are also nonspecific; thus, there is a risk that these patients might be misdiagnosed for another pathology. Paolini et al., for example, reported that patients with thoracic spine CPPD presented with left-sided chest pain [46]. In our review, the vast majority of patients for whom we have data on the presenting complaints presented with lower back pain. This is followed by myelopathic features [22-24], especially in the presence of cervical or thoracic spine pseudogout. The full details of the presenting complaints are available in Table 2 above.

Demographics and Distribution

The average age of the patients in our review was 66 years. This is expected, as it is mostly a pathology found in the elderly population [8,25,26], and our findings are similar to many other works available in this field.

The male-to-female ratio was 1:1.25. Interestingly, most of the other works reviewed also show a slightly greater female preponderance [27,28]. Notably, from an extraspinal perspective, there is no major sex predominance of pseudogout. However, attacks of acute pseudogout appear more commonly in men, whereas women more commonly present with the pseudoarthritis pattern of the disease [25]. In our study, the lumbar region was the most common region involved (50.1%), followed by the cervical region (33.7%) and finally the thoracic spine (16.2%). Previous systematic reviews have considered spinal CPPD in a specific segment of the spine, such as the lumbar or cervical spine [29]; however, to the best of our knowledge, no systematic review has addressed all segments of the spine, and there is a paucity of data on which part of the spine is more likely to be involved in spinal CPPD. Notably, other papers reported that the cervical spine is most affected. There is, however, nothing in the literature to explain why certain segments of the spine might be more susceptible to calcium pyrophosphate deposition or if there is any relationship with spine instability.

Crowned Dens Syndrome

Many case reports refer to CPPD deposition at the atlanto-axial joint and the cruciform ligament as 'crowned dens syndrome' [30-32]. Some researchers, however, exclude this from cervical spine CPPD, whereas other authors describe it as a prominent feature of cervical involvement in CPPD.

Comorbidities and Pathophysiology

Gout was a reported comorbidity in only four of the 153 patients included in this review [33-35]. It is known that gout and pseudogout may coexist in a single inflammatory effusion, and 20% of patients with CPPD will have hyperuricemia, one-fourth of whom will develop gout at some point. However, the incidence of spinal pseudogout is not well established. The pathophysiology of CPPD is not well understood. Working theories suggest that this phenomenon is due to the upregulation of extracellular inorganic pyrophosphate, which causes increased crystal formation secondary to underlying metabolic, posttraumatic, and hereditary etiologies.

Imaging Findings

The most common radiological feature present in patients in this review was degenerative changes. An epidural mass (not otherwise specified) was found in 13 patients, a calcified mass was found in 11 patients, and a cyst was found in seven patients. The full details are available in Table 4. The vast majority of patients in our review had an MRI with or without a CT scan as part of their radiological workup before surgery. Wu et al. [55] wrote a paper exploring the CT imaging features of spinal CPPD with some very helpful observations. They reported that in 48.9% of the cases studied (n=264 lesions), the characteristic imaging findings were disc calcification, posterior ligament calcification (40.5%), sacroiliac articular cartilage calcification (6.5%), and calcification of ligaments around the odontoid process (4.2%). This is an important line of inquiry and would probably need more investigations/validation to help arrive at a set of radiological diagnostic criteria for spinal CPPD.

Intraoperative Findings

Intraoperatively, the most common description of the gross operative findings in our review was a 'chalky white deposit,' usually enmeshed within the hypertrophied ligaments (e.g., Figure 2). Other findings included a "calcified mass," "cyst," "mass lesion (not otherwise specified)," and "gritty material." We have also provided histology slides from our case series in Figure 3.

In our case series above, we also reported the presence of "calcified ligamentum flavum," a "hemorrhagic cyst," an "extradural mass" firmly attached to the dura, "whitish and gritty connective tissue," and "white chalky material." We believe that spinal surgeons should keep these details in mind when performing spinal decompressive procedures, as they might indicate the presence of spinal CPPD. To what extent this would influence any adjunct treatment or postoperative care is a matter that requires further investigation. Notably, the prognosis of spinal canal decompression in patients with CPPD does not appear to be worse than that in patients with degenerative lumbar stenosis on the basis of some reports [37].

Limitations

This study has several important limitations. Our institutional case series includes only six patients from a single center, limiting generalizability, and CPPD was identified opportunistically rather than through systematic histopathological screening of all specimens. The true institutional incidence remains unknown. Our systematic review was not prospectively registered, and we did not perform a formal risk of bias assessment, though we justify this by the observational nature of all included studies. The 20-year publication cutoff, while justified by the evolution of diagnostic techniques, may have excluded relevant earlier observations.

The available evidence base consists predominantly of case reports and small series (81% of included studies), introducing substantial publication bias. Data completeness was poor, with clinical presentation unreported in 50.66% of cases and inconsistent reporting of radiological features, comorbidities, and long-term outcomes. Outcomes were typically qualitative rather than measured with validated instruments.

Most importantly, our study is purely descriptive without comparative analysis. We cannot determine whether CPPD presence causally affects surgical outcomes or whether findings would differ with medical management. The favorable surgical outcomes may reflect appropriate patient selection rather than benign disease biology.

These limitations mean our findings are hypothesis-generating rather than practice-changing. Prospective studies with systematic screening, standardized data collection, control groups, and validated outcome measures are needed to address these knowledge gaps.

Management of Spinal CPPD

CPPD is thought to be due to the immune response to the pathological presence of calcium pyrophosphate crystals within joints, causing acute or chronic inflammatory arthritis [38]. Diagnosis follows the traditional diagnostic triad of history, physical examination, and investigations, similar to many other pathologies [39].

CT scan findings may have the highest sensitivity, as alluded to above. Blood tests, such as a full blood count, C-reactive protein, serum urate levels, and a renal profile, may also have an adjunctive role in diagnosis [40]. However, when diagnostic uncertainty exists, and there is clinical concern that could affect surgical planning or when medical management is being considered for presumed CPPD, performing a discovertebral biopsy for definitive histopathological diagnosis may be warranted [41].

Treatment of spinal CPPD generally involves surgical decompression where there is a compressive mass, medical treatment failure, or another indication for surgery, as well as the use of adjuvant nonsteroidal anti-inflammatory drugs (NSAIDs) and steroids. Other pharmacological treatments involve the use of colchicine and an interleukin-1 (IL-1) inhibitor (anakinra). Spinal CPPD and gout are generally considered to be crystal diseases mediated by IL-1-driven processes; thus, management is aimed at tackling acute inflammation via the use of medications. There has also been an increasing focus on the use of biological agents such as IL-1β blocking agents. These include a monoclonal antibody against IL-1β (canakinumab), a recombinant IL-1 receptor antagonist (anakinra), and a dimeric fusion protein of the IL-1 receptor and the IL-1 receptor accessory protein (rilonacept). The researchers have demonstrated that calcium pyrophosphate crystals are capable of activating the inflammasome NLR-P3-caspase-1-IL-1β pathway present in innate immune cells such as neutrophils [55,56].

## Conclusions

Spinal CPPD is considered to be a rare pathology, but this is most likely because it is underdiagnosed and often misdiagnosed for other entities, such as infection or tumors. Through this review, we have tried to shed more light on its epidemiology, presenting features, imaging, and intraoperative findings. We have also provided a histopathology perspective and investigated the currently available modalities of care. Given the paucity of data on spinal CPPD, this work is intended to be hypothesis-generating and to establish a foundation for future prospective investigations.

Future studies should focus on establishing standardized diagnostic criteria combining preoperative imaging characteristics with intraoperative findings to improve recognition of spinal CPPD. Additionally, investigations into optimal medical management strategies for patients with concurrent CPPD and degenerative spine disease who may not require surgical intervention would be valuable.

## Appendices

Appendix 1

**Table 6:** PICO and search notes Full search strategy PICO: Patient, Intervention, Comparison, Outcome

Search Title	Concurrence of Calcium Pyrophosphate Deposition Disease (Pseudogout) in Patients Undergoing Surgery for Spinal Degenerative Disease
Population(s)	Adults 18 years and older with an incidental finding of pseudogout during surgery for spinal degenerative disease
Intervention(s)	Surgery for spinal degenerative disease, e.g, laminectomy or spinal instrumentation
Comparators	NA
Outcomes	Histological confirmation of the presence of spinal pseudogout or CPPD (calcium pyrophosphate deposition disease) in intraoperative samples, e.g., facet joint cysts or ligaments
Exclusion criteria	Non-English papers, patients less than 18 years, animal/cadaveric studies
Search period	The last 20 years

Appendix 2

**Table 7:** Search strategies

Databases Searched	Date of Search	Number of Results
Ovid MEDLINE(R) ALL <1946 to June 28, 2024>	01/07/2024	80
Embase <1974 to 2024 Week 26>	01/07/2024	185
Cochrane Central Register of Controlled Trials (CENTRAL) and Cochrane Database of Systematic Reviews (CDSR)	01/07/2024	CENTRAL: 6 CDSR: 0
Total results after deduplication	211 (after subtracting duplicates)	

Appendix 3

**Table 8:** Database

Database:	Ovid MEDLINE(R) ALL <1946 to June 28, 2024>	Results Per Line:	Number of Results:
Date:	01/07/2024		
1	Chondrocalcinosis/	2171	80
2	(calcium* adj4 (phosphate* or pyrophosphate*) adj4 (deposit* or crystal*)).ti,ab,kw,kf.	3741	
3	(CPP or CPPD).ti,ab,kw,kf.	14197	
4	(pseudogout* or chondrocalcinos* or crystal induced arthr* or crowded den).ti,ab,kw,kf.	2152	
5	1 or 2 or 3 or 4	19169	
6	exp Spinal Diseases/ or exp Spinal Cord Diseases/	287655	
7	((spine or spinal or vertebra* or intevertebra* or cervical spine or lumbar spine or thoracic spine) adj3 (disease* or disorder* or degenerat* or de-generat* or displace* or abnormalit* or malform* or anomal* or deform* or defect*)).ti,ab,kw,kf.	43076	
8	((spine or spinal or vertebra* or intevertebra* or cervical spine or lumbar spine or thoracic spine) adj3 (atroph* or compress* or h?ematoma or inflamm* or infarct* or infect* or lesion* or lipoma* or metastas* or necrosis or neoplasm* or plasmacytoma* or stenos?s or scoliosis or tumo?r*)).ti,ab,kw,kf.	70350	
9	(spondylit* or spondylos* or spondylarthrit*).ti,ab,kw,kf.	27090	
10	6 or 7 or 8 or 9	343885	
11	exp Spine/	170205	
12	(spine or spinal or vertebra* or intevertebra* or cervical spine or lumbar spine or thoracic spine).ti,ab,kw,kf.	601924	
13	11 or 12	655606	
14	exp Surgical Procedures, Operative/	3633556	
15	laminectomy/ or laminoplasty/ or spinal fusion/	42399	
16	(surger* or surgical* or operat* or intraoperat* or intra-operat* or perioperat* or peri-operat* or postoperat* or post-operat* or resect* or re-sect*).ti,ab,kw,kf.	3674581	
17	(laminectom* or laminoplast* or hemilaminectom* or facetectom* or instrumentat* or fuse* or fusion* or compress* or decompress* or de-compress*).ti,ab,kw,kf.	623812	
18	14 or 15 or 16 or 17	6183265	
19	13 and 18	229332	
20	5 and 10 and 19	132	
21	limit 20 to English language	120	
22	limit 21 to last 20 years	80	

Appendix 4

**Table 9:** Database 2

Database:	Embase <1974 to 2024 Week 26>	Results Per Line:	Number of Results:
Date:	01/07/2024		
1	pseudogout/	1359	185
2	(calcium* adj4 (phosphate* or pyrophosphate*) adj4 (deposit* or crystal*)).ti,ab,kw,kf.	4626	
3	(CPP or CPPD).ti,ab,kw,kf.	19129	
4	(pseudogout* or chondrocalcinos* or crystal induced arthr* or crowded den).ti,ab,kw,kf.	2897	
5	1 or 2 or 3 or 4	24983	
6	exp spine disease or exp spinal cord disease/	609710	
7	((spine or spinal or vertebra* or intevertebra* or cervical spine or lumbar spine or thoracic spine) adj3 (disease* or disorder* or degenerat* or de-generat* or displace* or abnormalit* or malform* or anomal* or deform* or defect*)).ti,ab,kw,kf.	54247	
8	((spine or spinal or vertebra* or intevertebra* or cervical spine or lumbar spine or thoracic spine) adj3 (atroph* or compress* or h?ematoma or inflamm* or infarct* or infect* or lesion* or lipoma* or metastas* or necrosis or neoplasm* or plasmacytoma* or stenos?s or scoliosis or tumo?r*)).ti,ab,kw,kf.	95445	
9	(spondylit* or spondylos* or spondylarthrit*).ti,ab,kw,kf.	40189	
10	6 or 7 or 8 or 9	646362	
11	exp spine surgery/ or exp spinal cord surgery/	120466	
12	(surger* or surgical* or operat* or intraoperat* or intra-operat* or perioperat* or peri-operat* or postoperat* or post-operat* or resect* or re-sect*).ti,ab,kw,kf.	4768574	
13	(laminectom* or laminoplast* or hemilaminectom* or facetectom* or instrumentat* or fuse* or fusion* or compress* or decompress* or de-compress*).ti,ab,kw,kf.	767271	
14	11 or 12 or 13	5341510	
15	5 and 10 and 14	265	
16	limit 15 to English language	243	
17	limit 16 to last 20 years	185	

Appendix 5

**Table 10:** Database 3

Database:	Cochrane Central Register of Controlled Trials (CENTRAL) and Cochrane Database of Systematic Reviews (CDSR)	Results Per Line:	Number of Results:
Date:	01/07/2024		
#1	MeSH descriptor: [Chondrocalcinosis] explode all trees	28	CENTRAL:6 CDSR:0
#2	((calcium* NEAR/4 (phosphate* or pyrophosphate*) NEAR/4 (deposit* or crystal*))):ti,ab,kw	53	
#3	((CPP or CPPD)):ti,ab,kw	1265	
#4	((pseudogout* or chondrocalcinos* or crystal induced arthr* or crowded den)):ti,ab,kw	52	
#5	#1 OR #2 OR #3 OR #4	1353	
#6	MeSH descriptor: [Spinal Diseases] explode all trees	6598	
#7	MeSH descriptor: [Spinal Cord Diseases] explode all trees	4511	
#8	(((spine or spinal or vertebra* or intevertebra* or cervical spine or lumbar spine or thoracic spine) NEAR/3 (disease* or disorder* or degenerat* or de-generat* or displace* or abnormalit* or malform* or anomal* or deform* or defect*))):ti,ab,kw	6630	
#9	(((spine or spinal or vertebra* or intevertebra* or cervical spine or lumbar spine or thoracic spine) NEAR/3 (atroph* or compress* or h?ematoma or inflamm* or infarct* or infect* or lesion* or lipoma* or metastas* or necrosis or neoplasm* or plasmacytoma* or stenos?s or scoliosis or tumo?r*))):ti,ab,kw	11165	
#10	((spondylit* or spondylos* or spondylarthrit*)):ti, ab, kw	4649	
#11	#6 OR #7 OR #8 OR #9 OR #10	26856	
#12	MeSH descriptor: [Surgical Procedures, Operative] explode all trees	174359	
#13	((surger* or surgical* or operat* or intraoperat* or intra-operat* or perioperat* or peri-operat* or postoperat* or post-operat* or resect* or resect*)):ti,ab,kw	412725	
#14	((laminectom* or laminoplast* or hemilaminectom* or facetectom* or instrumentat* or fuse* or fusion* or compress* or decompress* or de-compress*)):ti,ab,kw	59061	
#15	#12 OR #13 OR #14	489395	
#16	#5 AND #11 AND #15	6	
